# Clinical History of an Abdominal Tumor1Read at the 36th annual meeting of the Medical Association of Central New York, held at Auburn, September 22, 1903.

**Published:** 1904-01

**Authors:** Edward Judson Wynkoop

**Affiliations:** Syracuse, N. Y.


### Clinical History of an Abdominal Tumor.[1]

By EDWARD JUDSON WYNKOOP, M. D., Syracuse, N. Y.

IN presenting this history the effort is made to show what seemingly trivial abnormalities may exist without receiving proper attention and what grave results follow their neglect.

W. K., male, aged 13 months was admitted to the Women's and Children's Hospital, November 13, 1902, with the following history: father living and well. Mother living but not in good health, and since taking this history, mother has died of chronic meningitis. Two brothers living and well, at 8 and 22 years, respectively. One sister living and well at 3 years.

Patient was a full-term healthy child. Labor normal. Child weighed at birth 9 lbs. Had always been a bottle-fed baby. When 4 days old had convulsions. These convulsions would occur as often as once an hour and lasted from 20 to 30 minutes, sometimes longer. For three days these convulsions continued. After these ceased and until the child was 3 or 4 months old, he was dull and apathetic. During this period he lost in weight and weighed less at 4 months than when born.

From 4 months on he began to gain in weight. During this period, however, he had some gastrointestinal disturbance; teeth developed slowly and the child was very irritable. During the last two weeks of October, he was very restless and cried almost constantly, and early in November the family physician was called and found a fracture of the right femur, lower third. No history of an injury could be obtained. The following day the *x*-ray was used and diagnosis confirmed. The child's leg was placed in plaster-paris cast and a few days later, as mother was not in condition to properly attend to the child, both mother and child were sent to the hospital.

---

1. Read at the 36th annual meeting of the Medical Association of Central New York, held at Auburn, September 22, 1903.

*Physical Examination.*—Child fairly well developed but very anemic. Complexion resembled the cachexia of malignant disease. Weight not given. Temperature, 98.6. Pulse, 98. Head normal. Teeth regular, but dentition slow. Chest barrel-shaped. Rachitic rosary present. Abdomen prominent. Rachitic spine. In medium line just below umbilicus, a firm, hard tumor could be outlined with its base extending toward symphysis pubis. Tumor was about the size and shape of large egg. Skin freely movable over it. No fluctuation. Resembled to the touch a hard, firm growth. Tumor not freely movable. Marked phimosis present. Right thigh in plaster cast. Length of limbs equal. As the child had urinated freely and bowels had not moved it was decided to clear the intestinal tract and watch developments.

On leaving the hospital the family physician was consulted regarding the tumor. The only history obtainable from him was that the day the leg was placed in a splint the abdomen was unduly prominent, but this had always been the case with the child, so no particular attention was paid to it. November 14, child had slept during night fairly well, but at times seemed restless. Nourishment taken well. Bowels moved freely. Urine passed freely. Temperature and pulse about the same. Abdominal tumor unchanged. Examination of urine: specific gravity, low; alkaline, clear, slight trace of albumin present. No casts. In the afternoon Dr. Clifford Mercer saw the patient with me. Blood count, R. B. C., 2,480,000; W. B. C., 4,000.

From the location of the tumor it was evident that it was connected with the bladder and it seemed from the child's general appearance that a malignant condition might exist. A soft catheter failed to enter the bladder. A hard catheter was then tried but would not pass beyond the neck. At this time the marked phimosis was noted, but its relation to the abdominal tumor was not considered. Following the withdrawal of the catheter considerable urine was evacuated and the tumor seemed smaller but of the same consistency. It was now very evident that there was an obstruction to the flow of urine, probably at the neck of bladder. During the day the child had been restless and feedings were not taken as well as usual. No vomiting.

Dr. Jacobson and Dr. A. G. Doust saw the patient with me the next day. During the night the child had been restless and would not take food well. Temperature, 97.6. Pulse, 100. The abdominal condition was unchanged. It was decided to anesthetise the child at once and if possible introduce a catheter into the bladder. At this time Dr. Jacobson suggested the possibility of the tight foreskin being the cause of the trouble. After the child was anesthetised, a silver catheter at once entered the bladder; 7 to 8 ounces of clear urine escaped and the tumor subsided, leaving only a small hard lump just above the symphysis. Circumcision was at once done. The child rallied well from the anesthetic but began to fail during the evening and died about 9.30.

*Autopsy.*—November 16, 9 a. m., by Dr. H. S. Stensland. Length of body, 64 cm. Head not examined. Body fairly well developed; rather poorly nourished. Rigor mortis not apparent; no edema; well defined rachitic rosary; recent operation for phimosis. Peritoneal cavity: serosa smooth and glistening; appendix normal; mesenteric lymph nodes not enlarged; lesser peritoneal cavity and pancreas normal; bladder prominent, hard, enlarged and thickened. Organs all very pale. Pleural cavities: no adhesion, no fluid. Pericardial cavity contained a small amount of clear fluid. Heart valves, cavities and coronary arteries, normal. Myocardium uniform in color. Lungs pale and downy; posterior surface red and of firmer consistency. Spleen lymph nodules and trabeculæ distinct. Gastrointestinal tract mucosa pale.

Pancreas, normal. Liver, brownish red, smooth. No adhesions about gall-bladder. Kidneys: left, large and lobulated; right, small, 3 cm. long; ureters and pelvis, long and tortuous; adrenals, normal; bladder, thick walled and large; urethral orifices patent. Aorta, intima smooth. Organs of the neck and brain not examined. Right thigh, separated at lower epiphysis; during manipulation upper epiphysis separated; shaft of femur surrounded by recent bloodclot. Left thigh, femur normal; epiphysis not easily separated.

*Anatomical Diagnosis.*—Chronic hydronephrosis; chronic hydroureter; distension and hypertrophy of bladder; malformation of kidneys; rachitis; separation of lower epiphysis of right femur; blood-clot surrounding shaft of right femur.

"Hydronephrosis is a condition of over-distention by fluid of the pelvis of the kidney, caused by obstruction of the normal outflow of urine through the ureter or through the urethra. It may be congenital or acquired, permanent or intermittent, unilateral or bilateral."

There is usually a rapid atrophy of the kidney following the formation of a complete hydronephrosis and a portion if not all of the ureter is involved in the dilatation, according to the position and the completeness of the obstruction to the flow of urine. When the obstruction is complete rapid atrophy of the kidney follows and secretion ceases, whereas, if the obstruction be permeable to a small amount of urine, great dilatation is likely to take place; in such a condition hypertrophy of the parenchyma rather than atrophy is yet to be found. Congenital hydronephrosis when bilateral is rapidly fatal, the child seldom living longer than a few months.

The causes of congenital hydronephrosis are many, among them being imperforate ureter or urethra, congenital tumors, and the like. Acquired hydronephrosis may be caused by stricture of urethra, enlarged prostate phimosis tumors of neighboring organs, and like conditions. Hypertrophy of the bladder may or may not be present. Double hydronephrosis is generally associated with or results in such changes in the kidneys that the patients die during infancy, commonly in the first year. In the great majority of cases the condition is unrecognised. Some English authorities believe that an indication of this condition can be obtained from the persistent low specific gravity of the urine, alkaline or neutral reaction, and the presence in it of a small amount of albumin.

The treatment of this condition is surgical. When the condition has persisted for some time and marked destruction has resulted, the mortality is high. A great many cases die in a few hours, whether the operation be circumcision or simply cutting a stricture. The cause of death is usually shock or uremia.

In regard to the case reported, the cause of the convulsions might be due to the condition of the genitourinary tract and not the gastrointestinal as is usual. Though sedatives were used to control these convulsions, would apathy remain for some three months without some other cause? The neglected foreskin escaped the notice of most of us and only by one was it mentioned as a possible cause of the entire trouble. The cachexia was so marked that malignant trouble was seriously considered. The size, shape and character of the tumor did not resemble in anyway a simple distended bladder.

REFERENCES.

Power: Surgical Diseases of Children.
Holt: Diseases of Infancy and Childhood.
Keating: Cyclopedia of Diseases of Children.
Dennis: System of Surgery.

210 JAMES STREET.

